# Supplementary material for: Barriers to implementing patient safety incident reporting and learning guidelines in specialised care units, KwaZulu-Natal: A qualitative study
Source: PLoS One. 2024 Mar 8;19(3):e0289857. doi: 10.1371/journal.pone.0289857 (PMC10923419; doi:10.1371/journal.pone.0289857)
Supplement: S1 Appendix — (PDF) [file pone.0289857.s001.pdf]

SUPPORTING INFORMATION: 2

INTERVIEW GUIDE: FOCUS GROUPS DISCUSSION

- **OPERATIONAL NURSE MANAGERS**

Site code: Date:
Start:
End:

Demographics
Gender:
Employment status:

INTRODUCTION:

Greetings,

Thank you for your time and for agreeing to participate in this study. The information you are going to share will contribute towards improving the implementation of patient safety incident reporting and learning guidelines in specialised care units. This interview is recorded and will take approximately 45 minutes to 60 minutes. Thank you for giving me permission to record this interview session and I would like to assure you that your institution and your name are not divulged. The information you give will is strictly confidential and only accessible to me, as a researcher and my supervisor, and will be used for research purposes only. A special code has been created for this virtual interview to avoid intruders. Participation is voluntary and please feel free to withdraw at any time, there will be no negative repercussions.

Questions:	Topics:
Which elements in your daily nursing practice influence patient safety?	Culture of Patient Safety
Tell me about your perceived knowledge and implementation of PSI reporting and learning guidelines in your unit	Implementation of PSI guidelines
How are the PSI reporting and learning guidelines implemented? Support from the organisation, e.g. PSI safety committees?	Handling of the PSI process, including classification and severity of patient outcome
What are the barriers and facilitating factors associated with PSI reporting and learning implementation?	Existing improvement strategy

Thank you for your time and participation
