# Supplementary material for: Barriers to implementing patient safety incident reporting and learning guidelines in specialised care units, KwaZulu-Natal: A qualitative study
Source: PLoS One. 2024 Mar 8;19(3):e0289857. doi: 10.1371/journal.pone.0289857 (PMC10923419; doi:10.1371/journal.pone.0289857)
Supplement: S2 Appendix — (PDF) [file pone.0289857.s002.pdf]

## SUPPORTING INFORMATION 1

### INTERVIEW GUIDE: INDIVIDUALS INTERVIEWS

- **ASSISTANT NURSE MANAGERS,**
- **CONSULTANT MEDICAL DOCTORS**
- **MONITORING & EVALUATION MANAGERS**

Site code: Date:
Start:
End:

**Demographics**
Gender:
Employment status:

**INTRODUCTION**

Good day Sir/Madam

Thank you for your time and for agreeing to participate in this study. The information you are going to share will contribute towards improving the implementation of patient safety incident reporting and learning guidelines in specialised care units. The interview is recorded and it will take approximately 45 minutes to 60 minutes. Thank you for giving me permission to record this interview session and I would like to assure you that your institution and your name are not divulged. The information you give will is strictly confidential and only accessible to me, as a researcher and my supervisor, and will be used for research purposes only. A special code has been created for this virtual interview to avoid intruders. Participation is voluntary and please feel free to withdraw at any time, there will be no negative repercussions.

**Essential question.**

1. Tell me about your experience as a senior manager, in implementing the PSI reporting guidelines, in your department.

2. **Probing questions**

- How PSI guidelines are communicated to you and the staff?
- How are the PSIs handled and managed in your institution?
- What are the associated factors that may contribute to the poor implementation of PSI reporting guidelines by healthcare professionals?
- What factors do you consider essential for the effective implementation of PSI reporting guidelines for ensuring quality care delivery?

Thank you for participating in my study.
