# Supplementary material for: Barriers to implementing patient safety incident reporting and learning guidelines in specialised care units, KwaZulu-Natal: A qualitative study
Source: PLoS One. 2024 Mar 8;19(3):e0289857. doi: 10.1371/journal.pone.0289857 (PMC10923419; doi:10.1371/journal.pone.0289857)
Supplement: S3 Appendix — (DOCX) [file pone.0289857.s003.docx]

| TRANSCRIPT | CODES | SUBTHEMES | THEMES | REMARKS |
| --- | --- | --- | --- | --- |
| **Focus groups: Operational managers**  **Hospital A**  **Question: Which elements in daily nursing practice influence patient safety?**  **Participant 1:** We ensure that there's adequate staffing, making sure that this also uhh...skills mix is uhh.. is ensured, enough ICU trained nurses each day, seven days per the week during the day and during the night. | I ensure that there's adequate staffing, making sure that this also uhh..skills mix is uhh.. is ensured, enough ICU trained nurses each day |  |  | **Positive** |
| **Participant 3:** I may be short of two nurses, find that the nurses that are brought to you…they even tell you, " I have never nursed a ventilated patient... I've never seen…’ she doesn't understand.... she's never seen a ventilator. She's relocated from High care... Not that she's not willing to work... she is willing.... she may ask you if you have another patient maybe there is not ventilated.... Then you say…I will allocate you, but the sister next to you, is going to assist you. But now you are compromising two patients.... you are compromising her patient, and you compromising this patient as well. So incidents are going to occur. | I have never nursed a ventilated patient... I've never seen…’ she doesn't understand.... she's never seen a ventilator. She's relocated from High care.  I will allocate you, but the sister next to you, is going to assist you. But now you are compromising two patients.... you are compromising her patient, and you compromising this patient as well. | Inadequate knowledgeable staff  Lack of support by Operational Manager  Work overload | **Lack of management support at the ground level** | **Negative** |
| **Participant 4:** I just wanted to add in terms of training, ahh… there's been a lot of complaints raised, I know, it's maybe it's out of our control, but since they stopped to take or sending people for post basic training, so as you have a lot of people that have exited through retirement and some got transferred and some resigned, ehh ...the replacement ratio is not the same. So you you lose a skilled person and she gets replaced by maybe maybe no replacement at all. And these people that you've got with no experience they're willing to work but they're not sent for training. Because somehow there's been.... I understand there's been a change of curriculum so everything's is at a standstill for now. So we don't have enough, I mean... skilled people. | in terms of training, ahh… there's been a lot of complaints raised, I know, it's maybe it's out of our control, but since they stopped to take or sending people for post basic training, so as you have a lot of people that have exited through retirement and some got transferred and some resigned, ehh ...the replacement ratio is not the same. So you you lose a skilled person and she gets replaced by maybe maybe no replacement at all. And these people that you've got with no experience they're willing to work but they're not sent for training. Because somehow there's been.... I understand there's been a change of curriculum so everything's is at a standstill for now. So, we don't have enough, I mean... skilled people. | Lack of specialised training  Change of curriculum for training  Lack of skilled staff  Lack of experience  Increased Retirement  Brain drain  Increased staff turnover  Lack of staff retention  Delayed replacement | **Human resource constraints** | **Negative** |
