# Supplementary material for: Barriers to implementing patient safety incident reporting and learning guidelines in specialised care units, KwaZulu-Natal: A qualitative study
Source: PLoS One. 2024 Mar 8;19(3):e0289857. doi: 10.1371/journal.pone.0289857 (PMC10923419; doi:10.1371/journal.pone.0289857)
Supplement: Attachment — Submitted filename: PONE-D-23-22765_reviewer.docx [file pone.0289857.s004.docx]

PLOS ONE

Barriers influencing implementation of patient safety incident reporting and learning guidelines in specialised care units, KwaZulu-Natal: A qualitative study.

--Manuscript Draft--

| Manuscript Number: | PONE-D-23-22765 |
| --- | --- |
| Article Type: | Research Article |
| Full Title: | Barriers influencing implementation of patient safety incident reporting and learning guidelines in specialised care units, KwaZulu-Natal: A qualitative study. |
| Short Title: | Barriers influencing implementation of patient safety incident reporting and learning guidelines |
| Corresponding Author: | Thusile Mabel Gqaleni, M.D.  University of KwaZulu-Natal School of Nursing and Public Health Durban, KwaZulu-Natal SOUTH AFRICA |
| Keywords: | Patient Safety incidents; implementation guidelines; specialised care units; Critical care |
| Abstract: | Background  Globally, increased occurrences of Patient Safety Incidents have become a public concern. The implementation of Patient Safety Incidents incident reporting and learning guidelines is fundamental to reducing preventable patient harm. To improve the implementation of these guidelines in specialised care units in KwaZulu- Natal, the views of healthcare professionals were unearthed.  Aim  This study explores the healthcare professional views toward the implementation of Patient Safety Incidents reporting and learning Guidelines in specialised care units. Methods  A descriptive, explorative qualitative approach was used to collect qualitative data from healthcare professionals working in specialised care units. The study was conducted in SCUs of three purposely selected public hospitals in two districts of KwaZulu-Natal.  Group discussions and semi-structured interviews were conducted from March to May 2021. Content data analysis was performed using Tesch’s method of analysis process. Results  The main themes that emerged during data analysis were: Ineffective reporting system affecting the communication of PSI guidelines, Inadequate Institutional and management for the healthcare professionals, Insufficient education and training of healthcare professionals, and poor human resources affecting the implementation of PSI guidelines.  The findings highlighted that there were more major barriers to the implementation of PSI reporting and learning guidelines.  Conclusion  This study confirmed that PSI reporting and learning guidelines are still not successfully implemented in the specialised care units and the barriers to implementation were highlighted. For rigorous implementation in South Africa, the study recommends revised PSI reporting and learning guidelines, designed in consultation with the frontline healthcare professionals, consisting of standardised, simple -user-friendly reporting process as well as a better implementation strategy to guide the healthcare professionals. Continuous professional development programmes may play an important role in the facilitation of the implementation process. |
| Order of Authors: | Thusile Mabel Gqaleni, M.D. |
|  | Sipho Wellington Mkhize, PhD |
| Additional Information: |  |
| Question | Response |
| Financial Disclosure  Enter a financial disclosure statement that | Author: TMH Gqaleni Grant Number:129904  Name of the Funder: National Research Foundation |

| describes the sources of funding for the work included in this submission. Review the [submission guidelines](http://journals.plos.org/plosone/s/submission-guidelines#loc-financial-disclosure-statement) for detailed requirements. View published research articles from [*PLOS ONE*](http://journals.plos.org/plosone/) for specific examples.  This statement is required for submission and will appear in the published article if the submission is accepted. Please make sure it is accurate. | ERL: https://nrfsubmission.nrf.ac.za  The funders had no role in study design, data collection and analysis, decision to publish, or preparation of the manuscript. |
| --- | --- |
| Unfunded studies  Enter: *The author(s) received no specific funding for this work.*  Funded studies  Enter a statement with the following details:   - Initials of the authors who received each award - Grant numbers awarded to each author - The full name of each funder - URL of each funder website - Did the sponsors or funders play any role in the study design, data collection and analysis, decision to publish, or preparation of the manuscript? - NO - Include this sentence at the end of   your statement: *The funders had no role in study design, data collection and analysis, decision to publish, or preparation of the manuscript.*   - YES - Specify the role(s) played. |  |
| * typeset |  |
| Competing Interests  Use the instructions below to enter a competing interest statement for this submission. On behalf of all authors, disclose any [competing interests](http://journals.plos.org/plosone/s/competing-interests) that could be perceived to bias this work—acknowledging all financial support and any other relevant financial or non- financial competing interests.  This statement is required for submission and will appear in the published article if the submission is accepted. Please make sure it is accurate and that any funding | The authors have declared that no competing interests exist. |

| sources listed in your Funding Information later in the submission form are also declared in your Financial Disclosure statement.  View published research articles from  [*PLOS ONE*](http://journals.plos.org/plosone/) for specific examples. |  |
| --- | --- |
| NO authors have competing interests  Enter: *The authors have declared that no competing interests exist.*  Authors with competing interests  Enter competing interest details beginning with this statement:  *I have read the journal's policy and the authors of this manuscript have the following competing interests: [insert competing interests here]* |  |
| * typeset |  |
| Ethics Statement  Enter an ethics statement for this submission. This statement is required if the study involved:   - Human participants - Human specimens or tissue - Vertebrate animals or cephalopods - Vertebrate embryos or tissues - Field research   Write "N/A" if the submission does not require an ethics statement.  General guidance is provided below. Consult the [submission guidelines](http://journals.plos.org/plosone/s/submission-guidelines#loc-guidelines-for-specific-study-types) for detailed instructions. Make sure that all information entered here is included in the Methods section of the manuscript. | The University of KwaZulu-Natal’s Human Science Research Ethics Committee (HSSREC/00001651/2020), and the Directorate: Health Research and Knowledge Management Unit (NHRD Ref: Kz_202010_0240), granted ethical clearance before data collection. Permission was also obtained from the Chief Executive Officers, heads of department and Senior Nurse Managers. Verbal consent to participate and voices to be recorded was obtained from the participants prior to data collection. The participants were informed of their rights to withdraw from the study at any time, without any negative repercussions. Ethical letter of approval has been uploaded. |

| Format for specific study types  Human Subject Research (involving human participants and/or tissue)   - Give the name of the institutional review board or ethics committee that approved the study - Include the approval number and/or a statement indicating approval of this research - Indicate the form of consent obtained (written/oral) or the reason that consent was not obtained (e.g. the data were analyzed anonymously)   Animal Research (involving vertebrate animals, embryos or tissues)   - Provide the name of the Institutional Animal Care and Use Committee (IACUC) or other relevant ethics board that reviewed the study protocol, and indicate whether they approved this research or granted a formal waiver of ethical approval - Include an approval number if one was obtained - If the study involved *non-human primates*, add *additional details* about animal welfare and steps taken to ameliorate suffering - If anesthesia, euthanasia, or any kind of animal sacrifice is part of the study, include briefly which substances and/or methods were applied   Field Research  Include the following details if this study involves the collection of plant, animal, or other materials from a natural setting:   - Field permit number - Name of the institution or relevant body that granted permission |  |
| --- | --- |
|  |  |
| Data Availability  Authors are required to make all data underlying the findings described fully available, without restriction, and from the time of publication. PLOS allows rare exceptions to address legal and ethical concerns. See the [PLOS Data Policy](http://journals.plos.org/plosone/s/data-availability) and [FAQ](http://journals.plos.org/plosone/s/data-availability#loc-faqs-for-data-policy) for detailed information. | No - some restrictions will apply |

| A Data Availability Statement describing where the data can be found is required at submission. Your answers to this question constitute the Data Availability Statement and will be published in the article, if accepted. |  |
| --- | --- |
| Important: Stating ‘data available on request from the author’ is not sufficient. If your data are only available upon request, select ‘No’ for the first question and explain your exceptional situation in the text box. |  |
| Do the authors confirm that all data underlying the findings described in their manuscript are fully available without restriction? |  |
| Describe where the data may be found in full sentences. If you are copying our sample text, replace any instances of XXX with the appropriate details.   - If the data are held or will be held in a public repository, include URLs, accession numbers or DOIs. If this information will only be available after acceptance, indicate this by ticking the box below. For example: *All XXX files are available from the XXX database (accession number(s) XXX, XXX.*). - If the data are all contained within the manuscript and/or Supporting Information files, enter the following: *All relevant data are within the manuscript and its Supporting Information files.* - If neither of these applies but you are able to provide details of access elsewhere, with or without limitations, please do so. For example:   *Data cannot be shared publicly because of [XXX]. Data are available from the XXX Institutional Data Access / Ethics Committee (contact via XXX) for researchers who meet the criteria for access to confidential data.*  *The data underlying the results presented in the study are available from (include the name of the third party* | All relevant data are within the manuscript and its Supporting Information files. |

| *and contact information or URL).*   - This text is appropriate if the data are owned by a third party and authors do not have permission to share the data.   * typeset |  |
| --- | --- |
| Additional data availability information: | Tick here if the URLs/accession numbers/DOIs will be available only after acceptance of the manuscript for publication so that we can ensure their inclusion before publication. |

<https://www.editorialmanager.com/pone/download.aspx?id=33778514&guid=a547de80-87b3-4729-a378-12b75c669242&scheme=1>Manuscript	[Click here to access/download;Manuscript;PLOS ONE manuscript.docx](https://www.editorialmanager.com/pone/download.aspx?id=33778514&guid=a547de80-87b3-4729-a378-12b75c669242&scheme=1)

##### 1 Barriers influencing implementation of patient safety incident reporting and learning

2 **guidelines in specialised care units, KwaZulu-Natal: A qualitative study**
3 T.M.H Gqaleni ^1^, Sipho. W. Mkhize ^1^
4 ^1^School of Nursing and Public Health, College of Health Sciences, University of
5 KwaZulu-Natal, Durban, South Africa.
6 E-mail:[gqalenit@ukzn.ac.za](mailto:gqalenit@ukzn.ac.za). (TMH)

7

8

# 9 Abstract

#### 10 Background

11 Globally, increased occurrences of Patient Safety Incidents have become a public concern.
12 The implementation of Patient Safety Incidents incident reporting and learning guidelines
13 is fundamental to reducing preventable patient harm. To improve the implementation of
14 these guidelines in specialised care units in KwaZulu- Natal, the views of healthcare
15 professionals were unearthed.

#### 16 Aim

17 This study explores the healthcare professional views toward the implementation of Patient
18 Safety Incidents reporting and learning Guidelines in specialised care units.

#### 19 Methods

20 A descriptive, explorative qualitative approach, was used to collect qualitative data from
21 healthcare professionals working in specialised care units. The study was conducted in
22 SCUs of three purposely selected public hospitals in two districts of KwaZulu-Natal. Group
23 discussions and semi-structured interviews were conducted from March to May 2021.
24 Content data analysis was performed using Tesch’s method of analysis process.

#### 25 Results

26 The main themes that emerged during data analysis were: Ineffective reporting system
27 affecting the communication of PSI guidelines, Inadequate Institutional and management
28 for the healthcare professionals, Insufficient education and training of healthcare
29 professionals, and poor human resources affecting the implementation of PSI guidelines.
30 The findings highlighted that there were more major barriers to the implementation of PSI
31 reporting and learning guidelines.

##### 32 Conclusion

33 This study confirmed that PSI reporting and learning guidelines are still not successfully
34 implemented in the specialised care units and the barriers to implementation were
35 highlighted. For rigorous implementation in South Africa, the study recommends revised
36 PSI reporting and learning guidelines, designed in consultation with the frontline healthcare
37 professionals, consisting of standardised, simple -user-friendly reporting process as well as
38 a better implementation strategy to guide the healthcare professionals. Continuous
39 professional development programmes may play an important role in the facilitation of the
40 implementation process.

# 41 Introduction

42 Globally, increased occurrence of Patient Safety Incidents (PSIs) and near misses have
43 become a public concern, which includes South Africa [1]. Near misses are incidents or
44 situations that have the potential to cause harm but did not reach the patient due to timely
45 intervention, whereas Patient Safety Incident (PSI) is harm caused by medical
46 mismanagement, instead of the underlying disease[2]. Globally, unsafe care is one of the
47 top 10 leading causes of death in the world and up to 83% of harm is avoidable [3]. The
48 Institute of Medicine reveals death related to PSIs varies from 44 000 to 98 000 people each
49 year with high-cost implications [4-6]. According to Flott, Fontana (3), over 420 million
50 hospitalisations each year around the world resulted in nearly 43 million PSIs, in 2013. An
51 analysis by Organisation for Economic Co-operation and Development (OECD) found that
52 15 percent of all hospital costs in OECD nations are due to patient harm from PSIs, with
53 associated costs ranging between $1.4 to $1.6 trillion, each year [7]. According to Choi, Pyo
54 (8), Lu, Ko (9), improving the hospital organization atmosphere is essential to facilitate
55 useful communication of patient safety incidents.
56 In Swedish and Brazilian health services, barriers and facilitators that influenced patient
57 safety were identified and the relevance of a multifaceted system perspective on patient
58 safety problems and solutions was pointed out [10, 11]. According to [3, 9], patient safety
59 is a multifactorial concept, driven by a complicated array of technical, human and system
60 factors. Several studies have identified barriers influencing the implementation of PSIs
61 reporting in healthcare settings including specialised care units (SCUs). These includes
62 underreporting, punitive culture, lack of standardized reporting system, staff shortages, lack
63 of safety culture, poor processing of incident reports, inadequate engagement of healthcare
64 professionals, limited institutional support of incident reporting systems including
65 inadequate usage of evolving health information technology [1, 12-17].
66 In middle and low-income countries (LMICs), almost one-third of patients who suffered
67 PSIs died and four out of five of those incidences were preventable [18].	Moreover, 134
68 million PSIs occur in hospitals due to unsafe care, resulting in 2.6 million deaths, each year
69 (National Academies of Sciences and Medicine, 2018). Also, it is estimated that 75% of
70 PSIs occur in the LMIC contexts, during hospital care and 10-15% of total deaths are as a
71 result of poor quality care [3]. In South Africa, a study conducted in KwaZulu-Natal reveals
72 high percentage of serious PSIs (47%) indicating poor implementation of PSI reporting
73 guidelines and lack of improvement strategies [19]. While incidents reporting is considered
74 a cornerstone to patient safety culture and quality improvement in healthcare [20, 21], under-
75 reporting in hospitals worldwide is still a challenge[1, 22, 23].
76 In South Africa, the implementation of Patient Safety Incident Reporting and Learning
77 Systems guidelines, as recommended by KwaZulu-Natal Department of Health (KZN
78 DoH), in line with National Department of Health (NDoH 2017) has provided no clear
79 evidence of the reduction of PSIs and if these guidelines are adequately implemented in
80 SCUs. Gqaleni and Mkhize (17), affirmed that healthcare professionals’ perception of
81 knowledge of PSI reporting and learning guidelines was good, however, the perception of
82 the implementation was poor. This study further recommended a revised implementation to
83 improve the quality and safety of healthcare in (SCUs). For SCUs, to heighten the positive
84 performance and practices of PSIs reporting and learning guidelines, the views of healthcare
85 professionals needed to be unearthed. Therefore, the aim of the study was to explore and
86 describe the views of the senior healthcare professionals regarding the implementation of
87 Patient Safety Incident reporting and learning guidelines in specialised care units of
88 KwaZulu- Natal.

# 89 Materials and Methods

## 90 Study design and setting

91 A descriptive, explorative qualitative approach, was used to collect qualitative data from
92 healthcare professionals working in SCUs. The study was conducted in SCUs of three
93 purposely selected public hospitals in two districts of KwaZulu-Natal. Two hospitals (A
94 and C), situated in the eThekwini district, provide both secondary and tertiary services, and
95 the third tertiary hospital (B) is in the uMgungundlovu district. The settings consisted of
96 specialised care units that included both Critical Care Units (CCUs), where the most
97 critically ill patients were admitted as well as High Care Units, where the recovering
98 critically ill patients were transferred but still needed specialised care.

##### 99 Table 1: Inclusion and Exclusion criteria

| **Inclusion** | **Exclusion** |
| --- | --- |
| Participants who were Operational Nurse Managers | Participants who were not Operational Nurse Managers |
| Participants who were Assistant Nurse Managers | Participants who were not Assistant Nurse Managers |
| Participants who were Consultant Medical doctors | Participants who were not Consultant Medical doctors |
| Participants who were Monitoring & Evaluation Managers | Participants who were not Monitoring & Evaluation Managers |
| Working experience of more than 10 years in specialised care units | Participants who did not work in these units and had less than 10 years of experience. |
| Willingness to participate | Not willing to participate |

100

## 101 Population, recruitment and sampling

102 Sampling is a process of selecting a portion of the population to represent the entire
103 population, so that inferences about the population can be made [24]. The target population
104 for this study consisted of senior healthcare professionals, who were involved in the
105 implementation of PSI reporting and learning guidelines in the SCUs.	Therefore, the
106 participants that were purposely selected were had expertise and experience in SCUs
107 environments and had vast experience in the handling of PSI reporting and learning
108 guidelines. The focus group discussions sample was comprised of Operational Nurse
109 Managers (OMs). Individual interviews included Assisting Nurse Managers (ANMs),
110 Monitoring, and Evaluation Managers (M&E), and Consultant Medical doctors (CMD).

### 111 Recruitment strategy

112 Appointments were made by the researcher with the institutions’ Senior Nurse Managers
113 and Operational Nurse Managers and all the participants prior to data collection. The
114 researcher had a briefing session with the participants, prior to data collection, explaining
115 the aim of the study and its importance to research. Participants were informed that they
116 were not forced to participate and that they had a right to withdraw from the study even if
117 they had already given consent, without any negative consequences, thus ensuring voluntary
118 participation.

## 119 Data collection process

120 This study was conducted during COVID-19 and the researcher followed the protocols. The
121 data were collected virtually to ensure social distancing, which was reinforced in the
122 boardrooms, including the use of personal protective equipment by the participants. Data
123 was collected over a period of 3 months, from August to October 2021.	Information
124 guidelines guided the focus group discussions and individual interviews, which consisted of
125 topics and probing questions to elicit more detailed information from the participants. The
126 researcher prepared one essential question with a list of probing open-ended questions,
127 although the participants were encouraged to speak freely about the topic, to obtain all the
128 information required.
129 Focus groups discussions, which consisted of Operational Nurse Managers, working in the
130 selected settings, as they formed homogeneous participants. These participants were also at
131 the forefront of implementing the PSI reporting and learning guidelines. Individual
132 interviews consisted of senior participants which were heterogenous, i.e. Assisting Nurse
133 Managers, Monitoring, and Evaluation Managers, and Consultant Medical Doctors to solicit
134 more in-depth information. The probing question were influenced by the participants’
135 responses and the interviewer sought clarity to minimize bias and subjectivity. Data were
136 collected until saturation was reached, that is, no new information will be emerging.
137 Interviews were conducted and lasted between 45 minutes to 60 minutes, and this allowed
138 the participants to freely elaborate on their opinions on the phenomenon. Participants gave
139 consent to record all the interviews and the researcher transcribed the data verbatim.

### 140 Ethical considerations

141 The University of KwaZulu-Natal’s Human Science Research Ethics Committee
142 (HSSREC/00001651/2020), and the Directorate: Health Research and Knowledge
143 Management Unit (NHRD Ref: Kz_202010_0240), granted ethical clearance and
144 permission to conduct the study before data collection. Gatekeepers’ permission was also
145 obtained from the Chief Executive Officers, heads of department and Senior Nurse
146 Managers. A verbal consent to participate and to the recording of the interview voices were
147 obtained from the participants prior to data collection. Grove, Burns (25) asserted that the
148 researcher must comply with three principles as stated by the Belmont report, namely; the
149 principle of beneficence, the principle of human dignity, and justice. The participants were
150 informed of their rights to withdraw from the study at any time, without any negative
151 repercussions.

## 152 Data analysis

153 Content data analysis was conducted using Tesch’s method of the analysis process [24].
154 Transcribed interviews were read several times and descriptive words were identified for
155 the formulation of thematic statements and concepts that created meaning. Notes were made
156 with ideas that came to mind. Each interview was read through and the underlying meaning
157 behind the scripts was determined. Notes were made on transcripts during the reading
158 process. A list of concepts was made and similar topics were clustered together. These
159 clustered topics were then be mapped as major topics. Raw data comprising transcripts were
160 reviewed again in the light of identified topics.	The text was coded and organised.
161 Descriptive words were identified in the text and used to describe themes or topics. Related
162 topics were grouped together. A final decision regarding categories was made. Preliminary
163 analysis was made and excerpts from the data supporting the identified categories and
164 themes was coded and recoded. A reflective journal was used to form themes and sub-
165 themes.

### 166 Trustworthiness

167 During the research process, the researcher applied four criteria, to determine
168 trustworthiness, namely, credibility, dependability, confirmability and transferability [26].
169 **Credibility.** According to Polit and Beck (27) credibility refers to the confidence in the
170 truth of the data and its interpretations, that is, the data must reveal what it is searching for.
171 The researcher ensured that this study was conducted in an authentic manner and the
172 researcher had a prolonged engagement with the participants, during the data collection
173 process, which assisted in gaining an in-depth understating of the phenomena under study.
174 **Dependability**. In this study the researcher provided detailed information of the research
175 process, findings and recommendations, so that the other prospective researchers could
176 replicate the study to their context. According to Polit and Beck (27), findings of an inquiry
177 must be able to be repeated and replicated with the same participants in the same context.
178 **Confirmability**. Confirmability refers to objectivity, that is, the potential for congruence
179 between two or more independent people about data’s accuracy, relevance, or meaning [27].
180 In this study, the research report conveyed participants’ experiences and not the perspectives
181 of the researcher. Themes generated during data collection reflected the tone of the
182 participants.
183 **Transferability**. Transferability refers to the extent to which findings can be generalized
184 and applied in other settings or groups [27]. In this study the researcher was responsible for
185 providing a detailed account of the research process, findings and recommendations, so that
186 the other prospective researchers could evaluate the applicability to their context.

# 187 Results

## 188 Introduction

189 Data were collected from the purposively selected participants until data saturation was
190 reached. Data analysis was conducted manually using the eight steps of Tesch’s method of
191 analysis. The first section provides the participants’ demographics, as illustrated in Table 2
192 and the second section outlines the findings of the study, thoughts and feelings expressed
193 by the participants during the in-depth interviews. The names of the participating hospitals
194 and the participants were not divulged to protect their identities.

##### 195 Table 2: Demographics of the participants

**Hospital	Gender	Job level**

**Focus group discussions**

Hospital A	7 Females; 3 Males	Operational managers

Hospital B	8 Females; 2Males	Operational managers

Hospitals C	6 Females; 2Males	Operational managers

**Individual interviews**

Hospital A	1 Female	Assistant Nurse Managers

Hospital B	1 Male	Assistant Nurse Managers

Hospitals C	1 Female	Assistant Nurse Managers

Hospital A	1Female; 2 Males	Consultant Medical doctor

Hospital B	1 Female; 2 Males	Consultant Medical doctor

Hospitals C	1 Male	Consultant Medical doctor

Hospital A	1 Female	Monitoring and evaluation Manager

Hospital B	1 Male	Monitoring and evaluation Manager

Hospitals C	1 Female	Monitoring and evaluation Manager

196

197 Note: **FGD**- Focus group discussion
198 **OM-** Operational Nurse Manager
199 **ANM**-Assistant Nurse Manager
200 **M&E**- Monitoring and Evaluation managers
201 **CMD**- Consultant Medical doctor
202 A summary of themes and subthemes that emerged during data analysis is presented in Table
203 3.
204 **Table 3: Themes and subthemes**

**Theme	Description of a theme	Sub-themes**

1.Ineffective reporting system

Communication factors are

1.1 Tedious, long and not simple

affecting communication of PSI

associated with the

guidelines

communication of PSI reporting

1.2 Unclear PSI guidelines and

and learning guidelines as

policies

perceived by the healthcare

professionals

1.3 Lack of consultation at the

forefront level

1.4 Inadequate feedback to staff

and family

2. Inadequate Institutional and

management for the healthcare professionals

Institutional support relates to the

support received by healthcare workers within the institution

2.1 A hostile environment

characterized by hierarchical structure and red-tape

2.2 Punitive culture
2.3 Lack of management support to frontline healthcare workers
2.4 Lack of teamwork
3. Insufficient education and

training of healthcare professionals

Education and training refer to

both qualification and expertise of healthcare professionals

3.1 Inadequate training of

healthcare professionals on PSIs

3.2 Lack of knowledge on how to construct the report properly
3.3 Lack of formal training of healthcare professionals
3.4 Lack of formal training, in- house training, and continued professional development programme.

4. Poor human resources affecting	Human resources refer to the

4.1 Shortage of staff

the implementation of PSI

factors that the healthcare

4.2 Inadequate competent a skill

guidelines

professionals perceived as

mix

availability of competent staff to	4.3 High staff turnover and lack of

implement PSI guidelines

staff retention

4.4 Busy environment

205 The views of some of the participants are presented as direct quotations to support the
206 identified themes and sub-themes.

### 207 Theme 1: Ineffective reporting system affecting communication of PSI

208 **guidelines**
209 Communication factors are associated with the communication of PSI reporting and
210 learning guidelines as perceived by the healthcare professionals. Participants had a
211 perception that communication was ineffective and not user-friendly to implement PSI
212 reporting and learning guidelines. Most participants stated that the actual structure of the
213 reporting system makes it difficult for the healthcare professionals to effectively implement
214 the guidelines.

##### 215 Subtheme 1.1: Tedious, long and not simple

216 The participants indicated that the reporting process was long and intense, tedious and
217 laborious, hence other PSIs were not reported.
218 *“It is a long form…the new form is very intense, apart from the report that you’re going*
219 *to write…. not simple at all.”* ***(ANM1, Hospital A, Female, from Individual interview)***
220 *“The time that it takes to fill in the PSI forms and then the possibility of further meetings*
221 *down the line, it is essentially inconvenience from the interview process, a very*
222 *laborious, inconvenient process.”* ***(CMD1, Hospital C, Male, from Individual***
223 ***interview)***

##### 224 Subtheme 1.2: Unclear PSI guidelines and policies

225 The healthcare professionals also stated that the guidelines were not clear, confusing and
226 not standardised. This was considered a barrier to effective implementation of the
227 guidelines.
228 *“The guidelines are not particularly clearer on when an adverse event can be*
229 *considered as just expected as a consequence of the disease for a particular patient*
230 *versus a true PSI. Our government is using their own terminology which is*
231 *unfortunately not based on international standards.”* ***(CMD2, Hospital A, Male, from***
232 ***Individual interview)***
233 Some participants verbalised that there was a mismatch of the guidelines with the actual
234 work environment
235 *“Action plans and policies are nice and they are there, it's something that is there and*
236 *are nice to read, but when it comes to practicality of it*	*guidelines may look good on*
237 *paper, but they are not effectively implemented.”* ***(OM2, Hospital A, Female, from***
238 ***FGD)***

##### 239 Subtheme 1.3: Lack of consultation at the forefront level

240 Healthcare professionals also reported a lack of consultation at the forefront level and non-
241 clinical involvement during the development phase of PSI reporting and learning guidelines.
242 *“I was never consulted… I just received a directive from the DOH and my concern is*
243 *that the people that develop these guidelines are not clinical, they have no*
244 *understanding of what is happening on the ground, they have no medical training.”*
245 ***(CMD2, Hospital A, Male, from Individual interview)***
246 *“I didn't have any input, maybe from other managers. I just received a new form from*
247 *the province informing us what is required.”* ***(ANM2, Hospital A, Male, from***
248 ***Individual interview)***

##### 249 Subtheme 1.4: Inadequate feedback to staff and family

250 Inadequate feedback to staff and family was also identified by the participants as barrier to
251 the implementation of PSI reporting and learning guidelines. Lack of feedback was more
252 from the senior management and was more of a redress meeting. Partial family involvement
253 if a PSI has occurred, near misses are usually overlooked because it is perceived as harm
254 that did not reach the patient.
255 *“So, people might say okay, what do we need to disclose that to the family because it*
256 *was a near miss. It wasn't a miss. The patient didn't suffer any harm.”* ***(CMD1, Hospital***
257 ***C, Male, from Individual interview)***
258 *“I've never had feedback. And I've done multiple incident reports reporting over the*
259 *years, never, ever, ever, ever had…. you would expect documents and feedback. You*
260 *don't ever get any feedback from them. It is a waste of time because you get nothing*
261 *back. Then the next time something happens you go…. I'm not going to do it again*
262 *because what was the point?”* ***(CMD1, Hospital B, Female, from Individual interview)***

### 263 Theme 2: Inadequate Institutional and management for the healthcare

264 **professionals**
265 Most participants expressed a lack of support, especially from the senior management, a
266 disjuncture of the leadership from the challenges experienced by the frontline workers,
267 resulting in poor of teamwork.

##### 268 Subtheme 2.1: Punitive culture

269 Most participants reported that there is a punitive culture associated with the implementation
270 of PSI reporting and learning guidelines. This was characterised by fear of the negative
271 repercussions when healthcare professionals attempt to comply, therefore this led to
272 underreporting of most PSIs.
273 *“Sometimes this leads to disciplinary measures. Sometimes you say, I'm going to give*
274 *you a warning. And the PN will complain that I committed myself by reporting, and now*
275 *I must face the negative consequences. They even threaten to consult the unions.”*
276 ***(ANM1, Hospital B, Male, from Individual interview)***
277 *“Also, people have a view that it is punitive, in a way... so, I have seen different*
278 *departments threaten each other with PSIs… eh... so if you call something a PSI there*
279 *is still very much a mindset that this is an accusation... and it is even been recognised*
280 *between departments to sort of thresh out their politics….… been used between*
281 *departments as a sort as a blame. Even various people are using it almost*
282 *punitively…aggressively” (****CMD1, Hospital C, Male, from Individual interview)***

283

284

##### 285 Subtheme 2.2: A hostile environment

286 Participants described environment as hostile, characterized by hierarchical structure and
287 red-tape resulting reluctance in the implementation of PSI guidelines by the healthcare
288 professionals.
289 *“registered nurses that fear, they don't want to report that that's why they are under-*
290 *reporting, because that fear you don't have support. It's always like a threatening*
291 *environment. All they've learned is that they must keep quiet. And what if I get fired?”*
292 ***(CMD3, Hospital A, Female, from Individual interview)***
293 *“To me it’s like your call to the court where you're gonna sit and when they discuss this*
294 *incident you feel that I am... I am on the wrong…”* ***(OM1, Hospital B, Female, from***
295 ***FGD)***

##### 296 Subtheme: 2.3 Lack of management support to frontline healthcare workers

297 In addition, participants expressed a lack of guidance when trying to implement reporting
298 and learning guidelines.	Participants verbalised that an enabling environment is crucial,
299 therefore inadequate organisational resources, patient safety committees that are not fully
300 functional, lack of implementation strategies to guide healthcare professionals and learning
301 opportunities were regarded as barriers.
302 *“There is lack of support. Because... we are OMs, we work with staff, and we've got*
303 *challenges. You need somebody to listen to you, if you say this and this is happening.*
304 *Instead, you're blamed.... you are criticised... you... you… you really feel bad.”* ***(OM2,***
305 ***Hospital B, Female, from FGD***
306 *“no one's learning from the previous mistakes And there's always another excuse. You*
307 *just hit your head against the wall.”* ***(CMD1, Hospital B, Female, from Individual***
308 ***interview)***
309 Participants also verbalised that patient safety committees were not existing, and those that
310 were existing were at a development stage.
311 *“But from what I hear they just getting it up and running and getting some processes in*
312 *order. I think the first steps are happening there… I view it as the start of the process.*
313 *but I don’t know if we’ve quite got the systems at the unit level… we don’t have the unit*
314 *SOP or patient safety committees on how to deal with a PSI... it is more of an ad-hoc*
315 *base …”* ***(CMD2, Hospital A, Female, from Individual interview)***
316 *“I'm not aware of any Patient Safety committee that exists. I don't even know what*
317 *happens what the process is after I've handed the form as far as it goes from the ward*
318 *to the ANM… that's the point of the SOP. I don't know what happens after that, which*
319 *is bad as well, because we need to know the entire process… it's all… it's all very*
320 *haphazard”.* ***(CMD2, Hospital C, Male, from Individual interview)***

##### 321 2.4 Lack of teamwork

322 Participants reiterated that there should be a learning experience during the implementation
323 of reporting and learning guidelines, not to blame someone. Participants reported that a
324 teamwork approach between departments and collaboration with other institutions was
325 lacking.
326 *“We rely on nurses on the reporting of the PSI for formal reporting as such, because of*
327 *a busy environment.” (****CMD2, Hospital A, Male, from Individual interview).***
328 *“Although we morbidity and mortality meetings where we discuss the PSIs as staff,*
329 *there is still that lack with collaboration with other institutions, we hardly meet…I don’t*
330 *remember us meeting….so there is a gap there.” (****ANM1, Hospital B, Male, from***
331 ***Individual interview)***

332

### 333 Theme 3: Insufficient education and training of healthcare professionals

334 Participants reported that education and training associated with implementation of PSI
335 reporting and learning guidelines, was multifaceted. The education and training challenges
336 included the actual formal education of registered nurses, lack of training in specialisation
337 of critical care, inadequate in-service training and workshops, to empower the participants
338 on the guidelines. Furthermore, The PSI guidelines were not made, freely available and
339 common knowledge to all the disciplines of departments.

##### 340 Subtheme 3.1: Lack of formal training of healthcare professionals

341 Participants confirmed that some of the nurses that were allocated to the specialised units
342 did not have the formal training and the skill that is required in this environment.
343 *“In most cases we were not even sure of their training course, as they were training in*
344 *these schools …mushrooming everywhere, and now come in as ENAs and passed, they*
345 *now want to be PNs, but still behaving like ENs…running the ward and there is no*
346 *senior sister. You face these challenges, as they were the ENs recently and that affects*
347 *the quality of nurses we employ.”* ***(ANM2, Hospital A, Male, from Individual***
348 ***interview)***
349 *“People don't know how to identify PSIs are? How do you report…There's a total lack*
350 *of education here...? So, you can't blame the individuals if they weren't educated in*
351 *this…*	*and so I do think there's a major problem.”* ***(CMD 2, Hospital A, Female, from***
352 ***Individual interview***

##### 353 Subtheme 3.2: Lack of knowledge on how to construct the report properly

354 Furthermore, the participants indicated that the reporting system is not user-friendly with
355 terminology that was not easy to understand. Although the participants knew about the
356 classification of PSIs, the reporting process was complex.
357 *“I think the cause is a lack of knowledge of how to write a PSI... Lack of knowledge on*
358 *how to construct properly... how to report properly, the whole incident... it has to be*
359 *concise …it has to be to the point, so if it is not written properly, I send it back…so it’s*
360 *more time needed.”* ***(ANM1, Hospital A, Female, from Individual interview)***
361 *“Then I mean, I'll say to the doctors you must fill out an incident reporting form and*
362 *they'll go ...What?... that nobody knows what it is. What to do with it.”* ***CMD1, Hospital***
363 ***A, Male, from Individual interview)***

##### 364 Subtheme 3.3: Lack of formal training, in-house training, and continued professional

365 **development programme**
366 Participants further indicated that there was insufficient continued professional development
367 programme to empower the healthcare professionals to construct the report properly.
368 Participants expected to have a common knowledge for all healthcare professionals and to
369 be familiar with the guidelines.
370 *“The perception that the staff is not adequately trained to identify the PSIs and to*
371 *disclose to patients and relatives.”* ***(ANM2, Hospital A, Male, from Individual***
372 ***interview)***
373 *“I think…. even the more senior clinician struggled to decide what is the PSI and what*
374 *isn’t a PSI…ah... so, do you do label something a PSI”* ***(CMD1, Hospital C, Male, from***
375 ***Individual interview)***

##### 376 Subtheme 3.4: Inadequate training of healthcare professionals on PSIs

377 Some participants reported that some of the items that are in the PSI reporting and learning
378 guidelines were well understood. They explained that some of the PSIs were expected as
379 normal process of the underlying illness.
380 *“Sometimes some of the items that are been included in those PSIs are not particularly*
381 *appropriate to the ICU, because its normal to follow the normal pathology in ICU.”*
382 ***(CMD2, Hospital B, Male, from Individual interview)***

### 383 Theme 4: Poor human resources affecting the implementation of PSI

384 **guidelines**
385 Most participants confirmed that inadequate competent, knowledgeable and experienced
386 was a huge barrier to effective implementation of PSI reporting and learning guidelines. The
387 inadequate staff allocation was exacerbated during COVID-19 pandemic.

388

##### 389 4.1 Subtheme: Shortage of staff

390 Specialised units are busy by nature, and normally a ratio of 1:1, that is comprised of
391 adequate skills mix. Participants stated that it was impossible to achieve this ratio, therefore
392 affecting the implementation of guidelines and compromised quality patient care.
393 *“the... conditions that we working in are not allowing us to make sure can uhhh…. we*
394 *try and ensure, but there isn’t adequate staff that staff is adequate, I mean people who*
395 *are who work here every day, and who knows what is happening here and not people*
396 *that I will be orientating. But the situation is that instead of having staff in this ward to*
397 *work, according to the guidelines, or the policy we still get people from all over who*
398 *will come here not knowing exactly what to do. Shortage itself is a big issue*
399 *(emphatically)You get somebody that has just qualified as PN, she has never worked in*
400 *a ward, she's just new...new PN She hasn’t seen a ventilator she's seeing the ventilator*
401 *for the first time. She's seeing the monitor for the first time.”* ***(OM5, Hospital B,***
402 ***Female, from FGD)***

##### 403 Subtheme 4.2: Inadequate competent a skill mix

404 Participants also indicated that the staff that was allocated to their departments did not have
405 specialised qualifications in SCU environment.
406 *“You need staff that will understand what is happening…. we don't even have time to*
407 *orientate them. It's just orientation... You need staff that will understand what is*
408 *happening…. we don't even have time to orientate them. It's just orientation.”* ***(OM5,***
409 ***Hospital B, Female, from FGD)***

410

##### 411 Subtheme 4.3: High staff turnover and lack of staff retention

412 Moreover, some participants expressed their frustration on high staff turnover and lack of
413 staff retention
414 *“There is also an increase in the number of retiring staff and the management is not*
415 *recruiting for the vacant posts… they are not doing anything new. The staffing challenge*
416 *is there. It takes too long to replace staff and you must motivate why you need more*
417 *staff. Although there may be trained and experienced ICU nurses, they are not enough*
418 *for the number of days and you find yourself not achieving that ratio of 1:”* ***(ANM3,***
419 ***Hospital A, Female, Individual interview)***

##### 420 Subtheme 4.4: Busy environment

421 Participants indicated that they work in a very environment, looking after vulnerable
422 critically ill patients that require individual attention.
423 *“It is very difficult for staff, especially in this busy critical care where there is staff*
424 *shortages… to now follow up … because somebody will be having a patient…remember*
425 *here the ratio here is 1:1 or 1:2.”* ***(ANM3, Hospital A, Female, Individual interview)***

426

427	*“we work with sort of skeleton staff. Every day you wonder how people are going to*

428	*cope with more admissions.”* ***(OM6, Hospital B, Female, from FGD)***

429

# 430 Discussion

431 This study sought to explore the views of the healthcare professionals who were at senior
432 level, regarding the barriers and facilitators to implementation PSI reporting and learning
433 guidelines in SCUs. Participants highlighted that although there are stipulated guidelines,
434 they are not effectively executed by the health care professionals. They reflected more
435 barriers than facilitators which could be perceived as hindrance the implementation of these
436 guidelines. All participants acknowledged the importance of revised improvement strategies
437 to facilitate the rigorous implementation and improvement of quality patient care was
438 essential.

439

### 440 Theme 1: Ineffective reporting system affecting communication of PSI

441 **guidelines**
442 In this study, the participants indicated that the difficulty was more on the process of
443 communication of the PSI reporting and learning guidelines.	This was at multi-level,
444 amongst the individuals, departments, senior management and the way the whole system is
445 structured. Also, an organisational patient safety cultures must be cultivated to create an
446 atmosphere that is conducive to communication of these guidelines. Barriers that were
447 mostly mentioned by the participants included a laborious inconveniencing process, unclear
448 guidelines and policies, not simple and user-friendly, lack of consultation at the coalface
449 and lack of feedback [1, 10, 11]. Several studies confirm that clear, standardised effective
450 communication is key to effective implementation by the healthcare professionals. Although
451 participants in Hospital B demonstrated a positive attitude to the implementation of PSI
452 reporting and learning guidelines, as they stated that they were partially involved during the
453 consultative process, this was not the case in hospital A and C, as they indicated that they
454 were never consulted. The government plays a critical role in monitoring and evaluation of
455 the implemented PSI guidelines, therefore the lack of involvement of Department of health
456 in managing incident reporting means that they are not aware of patient safety issues in the
457 hospitals in their regions [28]
458 Lack of feedback to the staff and patients and uncertainty about what to report does not
459 encourage the healthcare professionals to use the reporting system effectively, hence the
460 participants indicated that there was a lot of undocumented PSIs [3, 11, 15]. Participants
461 also indicated that there is no learning that takes place, where healthcare professionals can
462 use the PSI as learning opportunity to prevent recurrences, as they learn from their mistakes.
463 It is important that healthcare professional do not rely on reporting incidents only as reports
464 often do not lead to positive change [11]. Participants also stated that the feedback to patients
465 and families is partial, only if it is a major event. Martos Algarra (29) affirms that patients
466 become first victims and the professionals involved turn into second victims and all of them
467 need adequate support.

### 468 Theme 2: Inadequate Institutional and management for the healthcare

469 **professionals**
470 Investing in patient safety culture can be viewed as building an organizational resource,
471 which is beneficial for both improving the care quality and protecting staff well-being [9].
472 There is a need for more effective transformational leadership to facilitate a culture of
473 patient safety incident reporting and effective implementation of PSI reporting and learning
474 guidelines [22]. In this study, participants explained an atmosphere that is hostile, punitive
475 and lack of support of the frontline healthcare workers from the management [11, 22]. Fear
476 of negative repercussion and litigation were the main barriers of implementation of PSI
477 reporting and learning guidelines [30]. According to Ontario (30), Lee, Wu (31), a non-
478 punitive,	confidential,	independent,	expert	analysis,	timely,	system-oriented,	and
479 responsive, was suggested, a hospital should particularly pay more attention to the
480 confidentiality of case data in the system, when establishing an incident reporting system,
481 to avoid disputes and enhance reporting intention. Lack of teamwork and collaboration with
482 other departments and institutions was also mentioned by the participants. A similar study
483 highlighted a lack of collaboration between physicians and other health care personnel
484 functioned as a barrier to improved patient safety due to perceived hierarchical differences.
485 [10]. Participants also stated that although they had risk management meeting to discuss
486 PSIs, there was a lack of guidance in the form of patient safety committees, implementation
487 strategy and standard operational procedure, that was of common knowledge. A similar
488 study highlighted that the availability and use of written protocols that provide structure for
489 their work, such as guidelines and standardized care plans, positively influence patient
490 safety [10]. According to Dhamanti, Leggat (28) Lack of commitment to and priority of
491 patient safety, the complexity of the bureaucratic structure, and a lack of systematic
492 partnership and collaboration are problems that need to be addressed by systematic
493 improvement.

### 494 Theme 3: Insufficient education and training of healthcare professionals

495 Insufficient education and training that was expressed by participants was multifaced.
496 Participants mentioned poorly qualified healthcare professionals with no skills of working
497 in a specialised care environment. Insufficient skills could act as a barrier to enhanced
498 patient safety. Being a newly graduated nurse was mentioned as a barrier, due to a general
499 lack of experience and the need for learning concerning many new aspects of work [10].
500 Also, there was a lack on how to identify PSIs, how to construct a written report, inadequate
501 in-service training. A similar study confirm that lack of knowledge about incident reporting
502 systems, and lack of understanding about what constitutes an error was reported as common
503 barrier [30]. The government plays a critical role in ensuring patient safety in health care
504 services as they are actively involved in designing the guidelines and development of
505 policies. Therefore, either the government or medical institute managers should collect and
506 analyse the information in the system, reduce the reoccurrence of medical errors through
507 education and training and improvement activities, and enhance the patient safety culture of
508 the hospital [28]

509

### 510 Theme 4: Poor Human resources affecting the implementation of PSI

511 **guidelines**
512 Participants reported the lack of human resources was not only insufficient number of the
513 staff allocated to the specialised care units, but skill mix was also lacking. The staff shortage
514 was due to increased number of the staff at a retirement age, staff resigning for better
515 employment renumeration, recruitment by other countries for competent staff. This brain
516 drain could be associated with a burnout syndrome, therefore poor implementation of PSI
517 reporting and learning guidelines. The specialised care units are characterised by a busy,
518 stressful environment, with increased to work overload, working with a skeleton staff might
519 lead to burnout [32].	Shortage of staff and increased workload was associated poor
520 implementation of the PSI reporting guidelines [9, 16, 28]. Mossburg and Himmelfarb (32),
521 affirm that healthcare managers can consider a simpler measure of this resource in ensuring
522 adequate staffing levels across all departments of the institution.

# 523 Strengths and limitations of the study

524 This study provided thought provoking findings as in-depth information was collected from
525 the interviews from the focus group discussions and individual interviews from participants
526 who were at the managerial positions and were actively involved in the implementation of
527 PSI reporting and learning guidelines. In addition, the purposive sample from the focus
528 group was homogenous, whereas the individual interviews were heterogenous, which
529 provided a rich information of experiences from a various healthcare professional. To the
530 researchers’ knowledge this is the first study to enlighten the barriers to implementation of
531 PSI reporting and learning guidelines by the healthcare professionals in South African
532 SCUs.	Furthermore, the study findings may inform the policy makers and healthcare
533 facilities to revise the PSI reporting guidelines, to be more relevant to the South African
534 context and devise better implementation strategy, to improve patient safety care. In
535 addition, the findings from this study have identified areas that need improvement in
536 patient’s safety, therefore its dissemination contributes to the cultivation of patient safety
537 culture worldwide. The study participants were limited to three major government hospitals
538 in KwaZulu-Natal, and this main limitation of the study, however the sample was
539 representative, therefore enough to address the research question. Also, most of the literature
540 that supported this study is more international, there is paucity of South African literature.

# 541 Recommendations

542 This study was conducted in one province, therefore further research is recommended to be
543 conducted in other provinces. The study research analysis and results can be used by global
544 and country stake holders to improve the implementation of PSI reporting and learning
545 guidelines, as per WHO recommendation. For rigorous implementation in South African
546 SCUs, the study recommends revised PSI reporting and learning guidelines that consist of
547 standardised, simple -user-friendly terminology as well as a better implementation strategy
548 to guide the healthcare professionals to implement the guidelines effectively. These
549 guidelines need to be designed in consultation with the frontline healthcare professionals by
550 the policymakers. Continuous professional development programmes may play an
551 important role in facilitating the implementation process. Education on PSIs reporting and
552 learning guidelines should be incorporated early in the curriculum of healthcare
553 professionals, so that it becomes common knowledge for all.

# 554 Conclusion

555 This study confirmed that PSI reporting and learning guidelines are still not effectively
556 implemented in the SCUs and the barriers of implementation were highlighted. There is a
557 need to implement these guidelines in middle and low-income countries, including South
558 Africa. The study revealed that ineffective reporting system, lack of institutional and
559 managerial support, insufficient education and training of healthcare professionals and poor
560 human resources were the main barriers to effective implementation of PSI and learning
561 guidelines. To effectively improve patient safety, this study seeks to strengthen
562 collaboration among the healthcare professionals, organisations, within the African
563 continent and globally.
564 **Supporting information**
565 **S1 Appendix: Interview guide for the focus group discussion**
566 **S2 Appendix: Individual Interview guide**

567

### 568 Acknowledgement

569 The authors would like to acknowledge the gatekeepers and the healthcare professionals
570 from the three hospitals, who assisted and participated in the study.

### 571 Author contributions.

572 **Conceptualization**: TMH Gqaleni, SW Mkhize
573 **Study design, data collection**: TMH Gqaleni.
574 **Formal analysis**: TMH Gqaleni.
575 **Investigation**: TMH Gqaleni.
576 **Methodology**: TMH Gqaleni.
577 **Project administration**: TMH Gqaleni.
578 **Resources**: TMH Gqaleni.
579 **Supervision**: SW Mkhize.
580 **Validation**: SW Mkhize.
581 **Writing – original draft**: TMH Gqaleni.
582 **Writing – review & editing**: TMH Gqaleni, SW Mkhize.

583

584	**Conflict of interest:** None

585

586

587

588

589

590

591

592

593

594

595

596

597

598

599

600

# 601 References

602 1.	Yali G, Nzala SH. Healthcare Providers’ Perspective on Barriers to Patient Safety
603 Incident Reporting in Lusaka District. Journal of Preventive and Rehabilitative
604 Medicine. 2022;4(1):44-52. https://doi: 10.21617/jprm2022.417
605 2.	WHO. National Guideline for Patient Safety Incident Reporting and Learning in
606 the Public Health Sector of South Africa. World Health Organization; 2017.
607 3.	Flott K, Fontana G, Darzi A. The global state of patient safety. London: Imperial
608 College London. 2019.
609 4.	James JT. A new, evidence-based estimate of patient harms associated with
610 hospital care. Journal of patient safety. 2013;9(3):122-8.
611 https:/[/www.jstor.or](http://www.jstor.org/stable/26633011)g[/stable/26633011](http://www.jstor.org/stable/26633011)
612 5.	Makary MA, Daniel M. Medical error—the third leading cause of death in the US.
613 Bmj. 2016;353. https:/[/www.jstor.or](http://www.jstor.org/stable/26942667)g[/stable/26942667](http://www.jstor.org/stable/26942667)
614 6.	Lt K. To err is human: building a safer health system. Institute of Medicine,
615 Committee on Quality of Health Care in America. 2000.
616 7.	National Academies of Sciences E, Medicine. Crossing the global quality chasm:
617 Improving health care worldwide: National Academies Press; 2018.
618 8.	Choi EY, Pyo J, Ock M, Lee S-i. Nurses' perceptions regarding disclosure of
619 patient safety incidents in Korea: a qualitative study. Asian Nursing Research.
620 2019;13(3):200-8. <https://doi.org/10.1016/j.anr.2019.05.002>
621 9.	Lu L, Ko Y-M, Chen H-Y, Chueh J-W, Chen P-Y, Cooper CL. Patient Safety and
622 Staff Well-Being: Organizational Culture as a Resource. International Journal of
623 Environmental Research and Public Health. 2022;19(6):3722.
624 <https://doi.org/10.3390/ijerph19063722>
625 10.	Ridelberg M, Roback K, Nilsen P. Facilitators and barriers influencing patient
626 safety in Swedish hospitals: a qualitative study of nurses’ perceptions. BMC
627 nursing. 2014;13:1-12. https://doi.org/10.1186/1472-6955-13-23
628 11.	Alves MdFT, Carvalho DSd, Albuquerque GSCd. Barriers to patient safety
629 incident reporting by Brazilian health professionals: an integrative review. Ciencia
630 & saude coletiva. 2019;24:2895-908. https://doi.org/10.1590/1413-
631 81232018248.23912017
632 12.	Archer S, Hull L, Soukup T, Mayer E, Athanasiou T, Sevdalis N, et al.
633 Development of a theoretical framework of factors affecting patient safety incident
634 reporting: a theoretical review of the literature. BMJ open. 2017;7(12):e017155.
635 https://doi: 10.1136/bmjopen-2017-017155
636 13.	Rashed A, Hamdan M. Physicians' and nurses' perceptions of and attitudes toward
637 incident reporting in Palestinian hospitals. Journal of patient safety.
638 2019;15(3):212-7. https://doi: 10.1097/PTS.0000000000000218
639 14.	Khoshakhlagh AH, Khatooni E, Akbarzadeh I, Yazdanirad S, Sheidaei A. Analysis
640 of affecting factors on patient safety culture in public and private hospitals in Iran.
641 BMC health services research. 2019;19(1):1-14. https://doi.org/10.1186/s12913-
642 019-4863-x
643 15.	Gqaleni TM, Bhengu BR. Adverse events reporting system as experienced by
644 critical-care nurses in Kwazulu-Natal, South Africa. Africa Journal of Nursing and
645 Midwifery. 2018;20(1):1-14. <https://doi.org/10.25159/2520-5293/2456>
646 16.	Martos Algarra C. The role of forgiveness in disclosure and victim suport after a
647 patient safety incident. 2022. <http://hdl.handle.net/10803/673273>
648 17.	Gqaleni T, Mkhize S. Healthcare professionals’ perception of knowledge and
649 implementation of Patient Safety Incident Reporting and Learning guidelines in
650 specialised care units, KwaZulu-Natal. Southern African Journal of Critical Care.
651 2023:25-30. . https://doi.org/10.7196/SAJCC.2023.v39i1.559
652 18.	WHO. Patient safety tool kit: World Health Organization. Regional Office for the
653 Eastern Mediterranean; 2015.
654 19.	Gqaleni TM, Bhengu BR. Analysis of Patient Safety Incident reporting system as
655 an indicator of quality nursing in critical care units in KwaZulu-Natal, South
656 Africa. Health SA Gesondheid. 2020;25(1):1-
657 8. <https://doi.org/10.4102/hsag.v25i0.1263>
658 20.	Dhamanti I, Leggat S, Barraclough S, Tjahjono B. Patient safety incident reporting
659 in Indonesia: an analysis using World Health Organization characteristics for
660 successful reporting. Risk management and healthcare policy. 2019:331-8.
661 <http://doi.org/10.2147/RMHP.S222262>
662 21.	ELMeneza S, AbuShady M. Anonymous reporting of medical errors from The
663 Egyptian Neonatal Safety Training Network. Pediatrics & Neonatology.
664 2020;61(1):31-5. <https://doi.org/10.1016/j.pedneo.2019.05.008>
665 22.	Yusuf Y, Irwan AM. The influence of nurse leadership style on the culture of
666 patient safety incident reporting: a systematic review. British Journal of Healthcare
667 Management. 2021;27(6):1-7. <https://doi.org/10.12968/bjhc.2020.0083>
668 23.	Gong Y. Challenges and Opportunities of Patient Safety Event Reporting.
669 Accident and Emergency Informatics. 2022:133-50.
670 https://doi:10.3233/SHTI220014
671 24.	Burns N, Grove SK, Gray JR. Understanding nursing research-eBook: Building an
672 evidence-based practice: Elsevier Health Sciences; 2015.
673 25.	Grove SK, Burns N, Gray J. The practice of nursing research: Appraisal, synthesis,
674 and generation of evidence: Elsevier Health Sciences; 2012.
675 26.	Shenton AK. Strategies for ensuring trustworthiness in qualitative research
676 projects. Education for information. 2004;22(2):63-75. DOI: 10.3233/EFI-2004-
677 22201
678 27.	Polit, Beck. Nursing research: Generating and assessing evidence for nursing
679 practice. Philadelphia: Wolters Kluwer| Lippincott Williams & Wilkins; 2017.
680 28.	Dhamanti I, Leggat S, Barraclough S, Liao H-H. Comparison of Patient Safety
681 Incident Reporting Systems in Taiwan, Malaysia, and Indonesia. Journal of patient
682 safety. 2021;17(4):e299-e305. DOI: 10.1097/PTS.0000000000000622
683 29.	Martos Algarra C. The role of forgiveness in disclosure and víctima suport after a
684 patient safety incident: Universitat Internacional de Catalunya; 2022.
685 <http://hdl.handle.net/10803/673273>
686 30.	Ontario HQ. Patient safety learning systems: a systematic review and qualitative
687 synthesis. Ontario health technology assessment series. 2017;17(3):1.
688 PMID: [28326148](https://pubmed.ncbi.nlm.nih.gov/28326148)
689 31.	Lee Y-C, Wu H-H, Weng S-J, Huang Y-C, Hsieh W-L, Huang C-H. Application of
690 Hospital Information Systems-Construction of an Incident Reporting System. TEM
691 Journal. 2016;5(4).
692 32.	Mossburg SE, Himmelfarb CD. The association between professional burnout and
693 engagement with patient safety culture and outcomes: a systematic review. Journal
694 of patient safety. 2021;17(8):e1307-e19.

695

696

Supporting Information

[Click here to access/download](https://www.editorialmanager.com/pone/download.aspx?id=33778544&guid=d33191fc-b3bf-4609-9750-5b68a5b2e062&scheme=1)

[Supporting Information Interview guide for Focus groups.pdf](https://www.editorialmanager.com/pone/download.aspx?id=33778544&guid=d33191fc-b3bf-4609-9750-5b68a5b2e062&scheme=1)

Supporting Information

[Click here to access/download](https://www.editorialmanager.com/pone/download.aspx?id=33778626&guid=5a5254b7-d0c1-4bdc-b3db-457a4f0f4556&scheme=1)

[Supporting Information Individual Interview guide.pdf](https://www.editorialmanager.com/pone/download.aspx?id=33778626&guid=5a5254b7-d0c1-4bdc-b3db-457a4f0f4556&scheme=1)
