# Supplementary material for: Barriers to implementing patient safety incident reporting and learning guidelines in specialised care units, KwaZulu-Natal: A qualitative study
Source: PLoS One. 2024 Mar 8;19(3):e0289857. doi: 10.1371/journal.pone.0289857 (PMC10923419; doi:10.1371/journal.pone.0289857)
Supplement: Attachment — Submitted filename: Response to Reviewers.docx [file pone.0289857.s005.docx]

PLoS ONE - 'Response to Reviewers'

| Reviewer #1: Can you please provide a explanation of the published article 'Gqaleni TM, Mkhize SW. Healthcare professionals’ perception of knowledge and implementation of Patient Safety Incident reporting and learning guidelines in specialised care units, KwaZulu-Natal. Southern African Journal of Critical Care. 2023 Mar 1;39(1):25-9.' and how this submission adds to what is already been presented in the article cited |  | This article is part of the PhD thesis by manuscript. The research approach was a mixed-methods |
| --- | --- | --- |
| Reviewer #2: The manuscript is well written and have scientific evidence, the results are well presented, and discussion of results is provided in a logic way and supported by literature.  The methodology needs to be described in full, how participants were recruited and consented to participate virtually.  Due to lockdown restriction how were interviews done, which virtual platforms were used and how were ethical issues handled with these platforms.  Again, field notes and observations during interviews.  The population was highlighted but the anticipated sample size was not indicated, when did data saturation occur?  The prolonged engagement is not clear, was it determined by time the duration of the interviews? | Thank you  Addressed in Lines 134-140  Addressed in Lines 186-193  Lines 208-210  Lines 167-69  Line 218-220 | Due to virtual interviews, the researcher took field notes during each interview, based on the voice and body language. A reflective journal was used to form themes and sub-themes.    Data were collected until saturation was reached, that is, no new information emerged. The actual sample size of participants was 41 and data saturation was reached at participant 18.  The interviews were conducted between 50 to 60 minutes which allowed enough time for participants to elaborate on their opinions on the phenomenon. |
| How was it done virtually, can you clarify. | Line 148-155 | Addressed |
| How was data confirmed, with participants or external peers? Describe. | Line 230-232 | After the data transcription, participants were contacted telephonically, to confirm that the information provided was captured accurately. Also, the supervisor concurred with what was transcribed |
| Reviewer #3: Thank you for inviting me to review this valuable work. The authors have done great work, from conceptualizing the topic to writing the manuscript. Below are some comments and feedback for your consideration: | Thank you very much |  |
| 1. In the abstract, the study timeframe was stated as from March to May 2021; however, line 123 mentions it as from August to October 2021. Please revise and adjust this inconsistency accordingly. | Line 156 | August to October 2021. |
| 2. In line 58, please add the reference number at the end of the sentence | Line 82 | Corrected, thank you |
| 3. Please review the entire manuscript and use either parentheses ( ) or brackets [ ] consistently for in-text reference citations. |  | Corrected throughout the manuscript |
| 4. Consider adding one or two sentences about Table 1 after line 110 and insert the table just before the recruitment strategy section. | Line 132-136 | **Inclusion and Exclusion criteria**  As per illustration in table 1, Operational Nurse Managers, Assistant Nurse Managers, Consultants, Monitoring and Evaluating Managers, who had more than ten years of SCU experience were included in the study. Participants who did not work in these SCUs and those unwilling to participate in the study were excluded. |
| 5. Lines 102 and 166-186 contain extra information that does not appear to be necessary for the manuscript. |  | The comment has been noted as suggested. |
| 6. Please clarify why participants provided verbal consent instead of written, electronic consent. | Addressed in lines 186-192 |  |
| 7. While the interviews were conducted virtually, specifying which platform was used would be beneficial. | Addressed in lines 148-155 |  |
| 8. Label Table 2 as "Basic Characteristics of Participants" and, if available, include the mean age and years of experience. | Edited in Line 246 | The mean age is not available as it was not recorded. The research was more focused on the level of experience |
| 9. For Table 3, please describe the themes in the text or the table, but not both. | Line 259 | Table 3 was deleted, as themes described in the text |
| 10. In lines 477 and 490, mention only the first author and add "et al." | Line 533 and 546 | Corrected |
| 11. In the conclusion, consider removing "middle and low-income countries." | Line 613 | to implement these guidelines globally. |
| **Abstract**  **Aim** | | |
| Senior health care professionals, below it is indicated as such. Maintain consistency | Line 30 | This study explores the senior healthcare professionals' views toward the implementation of Patient Safety Incident reporting and learning Guidelines in specialised care units. |
| **Introduction** | | |
| The reference technique, checl author guidelines and maintain brackets or square brackets. | Corrected throughout the manuscript |  |
| **Methods**  **Study design and setting** | | |
| Insert senior | Line 114 | senior healthcare professionals |
| **Data collection process** | | |
| Which virtual platform was used? How were ethics matters handled in terms of data management and recording. For individual interviews were they also virtual/ telephonic interviews. How was informed consent, written consent handled? | Line 148-155  Line173-175 | In this study, Skype for individual interviews was used and Zoom was used for the focus group discussions, to collect qualitative data. To ensure privacy, for example, for Skype, participants needed to sign into the interview individually, which prevented unwanted intruders, and for Zoom meetings, passwords were used and the waiting room, which allowed the host researcher to control who entered the video conference  The data analysis document was stored in the researcher’s personal computer which is password-protected. Back-up copies were stored in a password-protected memory stick. All data was only accessible to the researcher, research assistant, and the supervisor. |
| **Ethical considerations** | | |
|  | Line 182-183  Line186-193 | The researcher had a briefing session with the participants, before data collection, explaining the aim of the study and its importance to research. The principle of beneficence, the principle of human dignity, and justice were observed throughout the study. Information sheets and informed consent forms were emailed to participants. Participants had to sign informed consent which was emailed back to the researcher, before the data collection. Furthermore, verbal consent to participate and to the recording of the interview voices were obtained from the participants before data collection. Moreover, a record of verbal permission is on the researcher’s recording device. This was further recorded on the interview guides, consisting of the participants’ site code, date, start, and end of the interview. (Supporting documents are uploaded online during the submission process, including the Ethical clearance certificate). The participants were informed of their rights to withdraw from the study at any time, without any adverse repercussions |
| **Data analysis**  **Trustworthiness** | | |
| **Credibility:** How was it done virtually, can you clarify. | Line 218-220 | addressed |
| **Confirmability:** How was data confirmed, with participants or external peers? Describe | Line 230-232 | addressed |

| Reviewer’s comments | Line | How it was addressed |
| --- | --- | --- |
| Please ensure that your manuscript meets PLOS ONE's style requirements, including those for file naming |  | Checked |
| We note that the grant information you provided in the ‘Funding Information’ and ‘Financial Disclosure’ sections do not match.  When you resubmit, please ensure that you provide the correct grant numbers for the awards you received for your study in the ‘Funding Information’ section |  | This will be corrected during the uploading of the revised manuscript |
| In your Data Availability statement, you have not specified where the minimal data set underlying the results described in your manuscript can be found.  Upon re-submitting your revised manuscript, please upload your study’s minimal underlying data set as either Supporting Information files or to a stable, public repository and include the relevant URLs, DOIs, or accession numbers within your revised cover letter | It will be uploaded during the submission of the revised manuscript |  |
| Important: If there are ethical or legal restrictions to sharing your data publicly, please explain these restrictions in detail. Please see our guidelines for more information on what we consider unacceptable restrictions to publicly sharing data  Note that it is not acceptable for the authors to be the sole named individuals responsible for ensuring data access.  We will update your Data Availability statement to reflect the information you provide in your cover letter |  |  |
| 4. We note that you have stated that you will provide repository information for your data at acceptance. Should your manuscript be accepted for publication, we will hold it until you provide the relevant accession numbers or DOIs necessary to access your data. If you wish to make changes to your Data Availability statement, please describe these changes in your cover letter and we will update your Data Availability statement to reflect the information you provide | Thank you, I still maintain |  |
| 5. We note you have included a table to which you do not refer in the text of your manuscript. Please ensure that you refer to Table 1 in your text; if accepted, production will need this reference to link the reader to the Table. | Line 137 | Corrected |
| 6. Please review your reference list to ensure that it is complete and correct. If you have cited papers that have been retracted, please include the rationale for doing so in the manuscript text, or remove these references and replace them with relevant current references. Any changes to the reference list should be mentioned in the rebuttal letter that accompanies your revised manuscript. If you need to cite a retracted article, indicate the article’s retracted status in the References list and include a citation and full reference for the retraction notice. | References checked and are correct |  |
