# Supplementary material for: Barriers to implementing patient safety incident reporting and learning guidelines in specialised care units, KwaZulu-Natal: A qualitative study
Source: PLoS One. 2024 Mar 8;19(3):e0289857. doi: 10.1371/journal.pone.0289857 (PMC10923419; doi:10.1371/journal.pone.0289857)
Supplement: Attachment — Submitted filename: Response to Reviewers -3.docx [file pone.0289857.s006.docx]

PLoS ONE - 'Response to Reviewers'

| **Additional Editor Comments:** | **Numbered lines** | **How it was addressed** |
| --- | --- | --- |
| Title: It can be shortened to “Barriers to implementing patient safety incident reporting and learning guidelines in specialised care units, KwaZulu-Natal: A qualitative study” | Line 1 | Revised |
| Rephrase “senior healthcare workers” into “healthcare workers in specialized care units.” The use of “senior” is misleading as it was not defined. If you insist on keeping the term, define it and insert the criteria you used | Revised- line 30,34,114 | Revised |
| Remove the “exclusion criteria.” These were simply the reverses of the inclusion criteria. The participants cannot be included if they do not meet the inclusion criteria. If no other criteria were used to exclude included participants, stating the exclusion criteria is unnecessary. | Line -133-137 | Table for inclusion and exclusion criteria removed |
| **Comments to the Author** |  |  |
| 1. If the authors have adequately addressed your comments raised in a previous round of review and you feel that this manuscript is now acceptable for publication, you may indicate that here to bypass the “Comments to the Author” section, enter your conflict of interest statement in the “Confidential to Editor” section, and submit your "Accept" recommendation |  | Reviewer #3: All comments have been addressed  Reviewer #4: (No Response) |
| 2. Is the manuscript technically sound, and do the data support the conclusions? |  | Reviewer #3: Yes  Reviewer #4: Yes |
| 3. Has the statistical analysis been performed appropriately and rigorously? |  | Reviewer #3: Yes  Reviewer #4: N/A |
| 4. Have the authors made all data underlying the findings in their manuscript fully available? |  | Reviewer #3: Yes  Reviewer #4: No |
| 5. Is the manuscript presented in an intelligible fashion and written in standard English? |  | Reviewer #3: Yes  Reviewer #4: Yes |
| **6. Review Comments to the Author** |  |  |
| **Reviewer #3**: The authors have addressed all the comments properly. The only concern is that Table 2 has extra numbers not related to the table itself. Please revise accordingly. | Line 161-166  Line 246 | Abbreviations explained in the text and table 2, now table 1 was revised |
| **Reviewer #4:** | **Methods** |  |
| - Line 133 The rationale for only including senior members of the PSI reporting team should be included. For example, junior members may be more honest about PSI reporting issues as they are not in management and less likely to cover up. |  | This article is part of the PhD thesis by manuscript. The research approach was a mixed-methods. The first article was quantitative in nature and the results were from the junior healthcare workers i.e. Registered Nurses and Registrars. For this manuscript, a qualitative approach was followed to interview senior healthcare workers. |
| - Line 156 Data collection process should describe the topic guide used - was a conceptual framework used? Or a list of topics explored should be given. Language and translation process should also be described if non-English language was used. | Line 592-593 | The data collection tools used were attached submitted with the manuscript. The conceptual framework was not used. The tools had the interview guides with topics, as well as the probing questions. English language was used as the healthcare workers agreed to be interviewed in English. |
| - Line 211 Trustworthiness- Suggest to remove this section and any elements felt important can be embedded in the methodology sections above. | Line 194-219 | Removed to the methodology section |
| - Table 1 can be revised to remove the exclusion criteria column, as reversing the inclusion is not useful. | Line 132 | Table removed |
|  | **Results** |  |
| - Line 254-258 , these abbreviations should be included in the text at first use, or embedded into Table 2. | Line 246 | Abbreviations embedded into the table |
| - In reporting quotes, suggest to remove whether it was an FGD or IDI, as it is known that OMs are FGDs and also it is not necessary to know if the quote came from FGD or IDI.  Otherwise it is noted that the author has made revisions based on prior reviewer comments which have been adequately addressed. |  | Corrections made throughout the document  That is correct |
| **7. Do you want your identity to be public for this peer review?** For information about this choice, including consent withdrawal. |  | Reviewer #3: **Yes:** Mona Abdelrehim  Reviewer #4: No |
